# Optical Sensor for Characterizing the Phase Transition in Salted Solutions

**DOI:** 10.3390/s100403815

**Published:** 2010-04-14

**Authors:** Rémy Claverie, Marc D. Fontana, Ivana Duričković, Patrice Bourson, Mario Marchetti, Jean-Marie Chassot

**Affiliations:** 1 Laboratoire Régional des Ponts et Chaussées de Nancy, Centre d’Études Techniques de l’Équipement de l’Est – 71 rue de la grande haie, 54510 Tomblaine, France; 2 Laboratoire Matériaux Optiques, Photonique et Systèmes, University Paul Verlaine of Metz and Supélec – 2 rue Edouard Belin, 57070 Metz, France; E-Mails: marc.fontana@metz.supelec.fr (M.D.F.); bourson@metz.supelec.fr (P.B.)

**Keywords:** Raman sensor, phase transition, salted solution

## Abstract

We propose a new optical sensor to characterize the solid-liquid phase transition in salted solutions. The probe mainly consists of a Raman spectrometer that extracts the vibrational properties from the light scattered by the salty medium. The spectrum of the *O* – *H* stretching band was shown to be strongly affected by the introduction of NaCl and the temperature change as well. A parameter *S_D_* defined as the ratio of the integrated intensities of two parts of this band allows to study the temperature and concentration dependences of the phase transition. Then, an easy and efficient signal processing and the exploitation of a modified Boltzmann equation give information on the phase transition. Validations were done on solutions with varying concentration of NaCl.

## 1. Introduction

Raman spectroscopy (RS) is a well-established technique to study the vibrational properties of solid, liquids or gas, in relation with the structure and the properties of the substance. It is less frequent to use RS as a probe of physical characteristics or properties of a substance. Raman sensors are able to employ recent technical improvements in the development of smart apparatus, with higher spatial resolution and possibilities of long-distance or on-line measurements [1,2].

One of the main advantages of a Raman sensor is the combination of the determination of a physical parameter, as in usual sensors, with the physical microscopic mechanism associated with its change. An additional advantage of the Raman probe is its non-destructive character, as many optical techniques. Furthermore in contrast with many other ones, it does not need any preparation of the specimen, allowing an on-site measurement, and only a small volume of the substance (diameter of one micrometer) is necessary for the analysis. It is reminded that the RS results in an inelastic collision between the exciting light beam with the substance under study and the energy shift of the photons provides the energy (frequency) of the optical phonons characterizing the substance.

The efficiency of the Raman effect and thus the scattered light intensity depends on the deformability and polarizability of chemical bonds. A Raman line is often specific to a chemical bond. Therefore RS can be used to identify the vibrational mode and thus the associated chemical bond responsible, as an example, to a phase transition. Three spectral parameters can be usually derived from the treatment of a Raman line. Generally the line is fitted to a lorentzian or to a gaussian shape, from which are deduced the peak maximum (phonon frequency shift), the FWHM (Full Width at Half Maximum) (or phonon damping) and the intensity. The phonon frequency is sensitive to any external parameter such as the temperature, the pressure, *etc.* affecting the substance. The linewidth of the Raman peak reflects the ordered or disordered character of the structure. At last, the intensity at the peak maximum, or rather the integrated intensity of the Raman line can be related to the concentration of a particular species in a material. As a consequence, the peak position (mode frequency), the linewidth (damping) and intensity extracted from a Raman line can be used for the determination of some physical parameters. The choice of the relevant parameter among these three possibilities mainly depends on the efficiency, the resolution and the accuracy which can be achieved using frequency, damping or intensity. In addition, it is generally possible to identify which chemical bond is responsible to a peculiar property of the substance and/or is affected by a change of an external parameter. Therefore, a Raman line can be considered as a real fingerprint of a chemical bond. The study detailed hereafter shows that the Raman spectrometry can be applied to the temperature and concentration dependences of the solid-liquid phase transition in a salted solutions.

## 2. Physical background of the sensor

**Figure 1.**
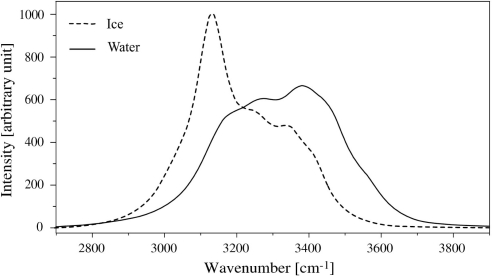
Typical Raman spectra of water at liquid and solid states.

**Figure 2.**
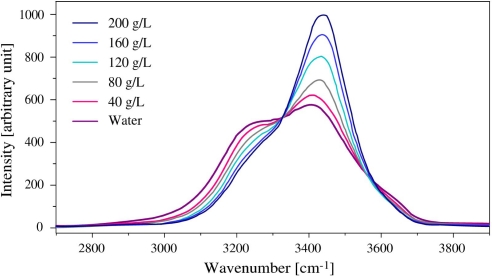
Concentration effect of the *O* – *H* stretching region on a Raman spectrum at 20 °C.

On Figure 1 is plotted the typical *O* – *H* stretching band recorded by RS in pure water in both solid and liquid phases. This broad band was widely investigated [3–5], and it is worth noting that the large change in its shape which occurs in both sides of the solid-liquid phase transition. This means that the lower part of the spectrum, which is related to fully *H* bonded atoms, is closely linked to the ordered solid phase, whereas the upper part which is ascribed by partly *H*−bonded and free *O* – *H* characterizes the liquid phase [6]. Figure 2 shows the same part of the spectrum recorded in NaCl liquid solutions of various concentrations. This clearly reflects the own influence of NaCl introduction which affects the higher frequency part.

From these observations, a parameter *S_𝒟_* is defined as the ratio of the lower and upper parts of the *O* – *H* spectrum, which should be able to describe the temperature and concentration dependences of the solid-liquid phase transition. The order or disorder character of the transition in salted solutions can be reflected from the values of this quantity. We propose to use this parameter deduced from Raman scattering measurements to study the phase transition characteristics. According to the frequency range of the spectrum recorded in pure water (see Figure 2), the acquisition of the spectrum is made from 3000 to 3650 cm^−1^. The *O* – *H* vibrations are then studied by comparing via *S_𝒟_* the integrated intensity *J*_1_ (3000 → 3325 cm^−1^) of the left side which is more sensitive to the temperature change and the integrated intensity *J*_2_ (3325 → 3650 cm^−1^) of the right side which is rather affected by the salt introduction. We therefore define the *S_𝒟_* parameter on Equation 1.

(1) $$S_{𝒟}=\frac{J_{2}}{J_{1}}$$

**Figure 3.**
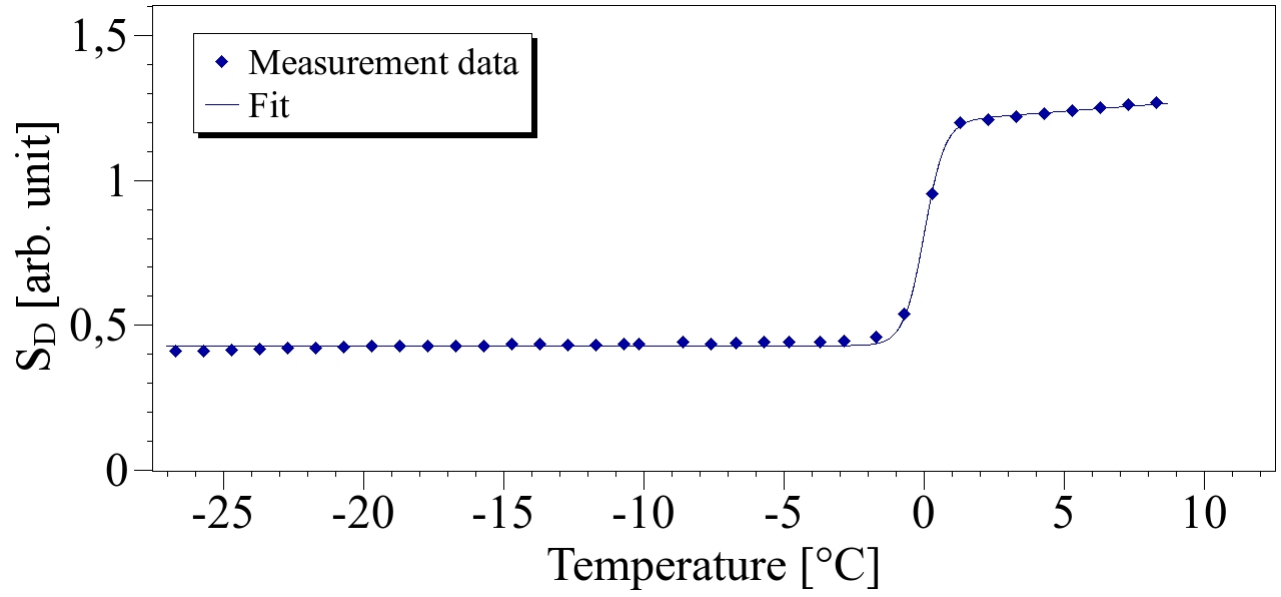
Points: data of pure water – Line: Fit with the modified Boltzmann equation (Equation 2).

The RS of *O* – *H* of water or brines is rather centered on 3325 cm^−1^. This value was shown as the limit between the right and the left frequency parts of the *O* – *H* spectrum. We have checked that such a choice of the exact limit is not so important. In fact the analysis of physical mechanisms involved in the *O* – *H* spectrum is more complicated and usually the spectrum is deconvoluted into 4, 5 or 6 bands to interpret it. This is the subject of many controversies [3,7,8]. Here we choose an easier way by considering only two parts which are rather linked to one or another process. The results described below show that the results derived from the method described here are fairly good. Figure 3 shows the typical plot of *S_𝒟_* for pure water from −25 °C to 10 °C. This material could therefore be considered clearly as a standard, its freezing point identified at 0 °C. We point out the phase transition around the mid part of the two extrema.

This plot can be, as usually, fitted by means of a modified Boltzmann equation (Michel [9] and Polidori [10]) shown on Equation 2.

(2) $$S_{𝒟}=(A_{2}+A_{3}\;\cdot \;T)+\frac{A_{1}-(A_{2}+A_{3}\;\cdot \;T)}{1+e^{(T-T_{c})/\Delta T}}$$

where *T* is the temperature of the medium, *T_c_* the phase transition temperature, *A*_1_ is the value of *S_𝒟_* when *T* ≪ *T_c_*, *A*_2_ is the y-intercept for the linear part of *S_𝒟_*, obtained for *T* ≫ *T_c_*, *A*_3_ is the slope for *T* ≫ *T_c_*, and Δ*T* is the specific slope at *T* = *T_c_*. Thus defined, the parameters *A*_1_ and *A*_2_ characterize the order and disorder process within the phase transition since they correspond to ice and liquid water, respectively. Indeed *A*_3_ is related to the thermal agitation, which is responsible for the breakdown of some hydrogen bonds, leading to an increasing disorder as the temperature raises.

As *S_𝒟_*, *A*_1_, *A*_2_ and *A*_3_ are quantities without units. We can notice that in case of *A*_3_ = 0, typical Boltzmann curve is well obtained.

The fit curve on Figure 3 is obtained by the Levenberg-Marquart algorithm on the −25 → +10°C temperature range and the regression coefficient is *R*^2^ = 0.998. It clearly indicates the good agreement between the experimental data and the calculated curve. From the results of the pure water, the suggested method was extended to study the phase transition characteristics of salted solutions of various concentrations.

## 3. Description of the probe and discussion of the results

**Figure 4.**
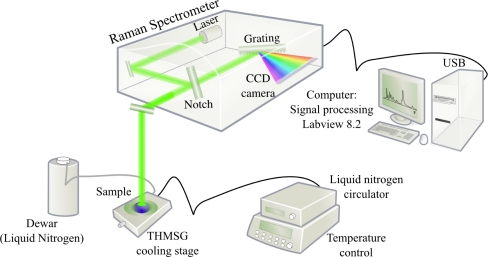
Materials of the sensor: the computer drives the spectrometer and proceed acquisitions and signal processing.

Figure 4 shows the sensor who mainly consists of a Raman spectrometer (including the laser, the notch filter and the CCD camera) and a computer for signal processing. The water sample is excited by an argon-ion laser at a wavelength of 514.5 nm, and with an output power of 25 mW. The Raman spectrum is measured in the backscattering configuration through a 50× long-working distance objective, located at about 8 mm of the top of the sample. The backscattered radiation is collected by a CCD camera of 1024 pixels.

The spectrometer with a spectral resolution of 2 cm^−1^ is linked to the computer by an USB plug. The software was developed to do acquisitions, calibrations and process the signal to calculate *S_𝒟_*, and to extract the phase transition temperature *T_c_*. The value *S_𝒟_* is calculated by midpoint rule and developed on Equation 3.

(3) $$S_{𝒟}=\frac{\sum_{i=326}^{650}\left[\frac{I(i)+I(i-1)}{2}\times \{n(i)-n(i-1)\}\right]}{\sum_{i=1}^{325}\left[\frac{I(i)+I(i-1)}{2}\times \{n(i)-n(i-1)\}\right]}$$

The main advantage of this simple method is the reduction of the noise influence. This calculus and the Levenberg-Marquart algorithm have been coded under LabVIEW 8.2.

In order to achieve to a simpler equation than Equation 2, calibrate of our sensor was performed by calculating the 5 parameters *A*_1_, *A*_2_, *A*_3_, *T_c_* et Δ*T* for any concentration. This technique also permits to save a significant calculation time. We perform several acquisitions of *S_𝒟_* for different concentrations in order to apply the modified Boltzmann regression.

**Figure 5.**
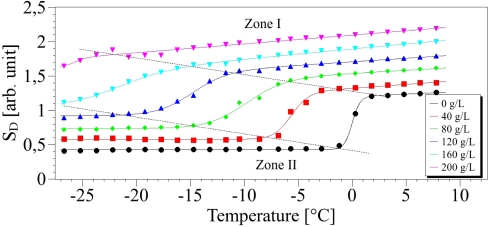
Points: concentration index *S_𝒟_* according to the temperature for several values of concentration. Lines: fit with Equation 2.

Figure 5 shows results of experimental data and associated fit for concentrations of salt from 0 to 200 g/L.

**Figure 6.**
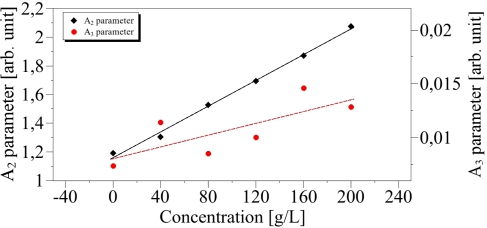
Linear relationship between the parameters *A*_2_ and *A*_3_ and the concentration.

The values of *A*_2_ and *A*_3_ of Equation 2 can be determined from the zone I of Figure 5 and are deduced from a linear relationship: *A*_2_ + *A*_3_ · *T* which derives from equation 2 when *T* ≫ *T_c_*. Both parameters are reported in Figure 6 as function of the concentration. We can observe on Figure 6, that a linear dependence of *A*_2_ and *A*_3_ on the concentration especially for *A*_2_. The standard errors are *s* = 0.02 and *s* = 0.29 respectively and the correlation coefficients are respectively *R*^2^ = 0.99 and *R*^2^ = 0.57. These values for *A*_3_ can be explained by the small slope of *S_𝒟_* leading to increasing uncertainties.

The relationship of *A*_2_ and *A*_3_ are then:

(4) $$\begin{array}{l} A_{2}=1.16+4.50\times 10^{-3}\;\cdot \;C\\ A_{3}=8.04\times 10^{-3}+2.74\times 10^{-5}\;\cdot \;C \end{array}$$

where *C* is the concentration. In the liquid phase, the behaviour of *S_𝒟_* can be linearly linked to the temperature as detailed Equation 5.

(5) $$S_{𝒟}=1.16+(4.50\times 10^{-3}+2.74\times 10^{-5}\;\cdot \;T)\;\cdot \;C+8.04\times 10^{-3}\;\cdot \;T$$

One should mention that temperature in pure liquid water could be therefore theoretically determined by Equation 5 with *C* = 0 g/L (see Equation 6).

(6) $$T=124.3\;\cdot \;S_{𝒟}-144$$

The liquid-solid phase transition zone was used to determine the *T_c_* and Δ*T* parameters defined in Equation 2, and their concentration dependences.

We obtained:

(7) $$\begin{array}{l} T_{c}=-0.131\;\cdot \;C\\ \Delta T=0.415+0.012\;\cdot \;C \end{array}$$

**Figure 7.**
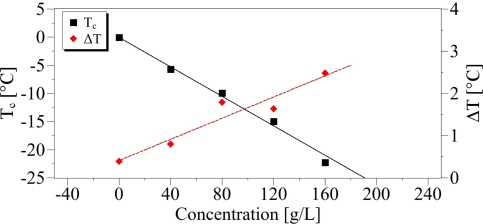
Linear relationship between the parameters *T_c_* and Δ*T* with the concentration.

**Figure 8.**
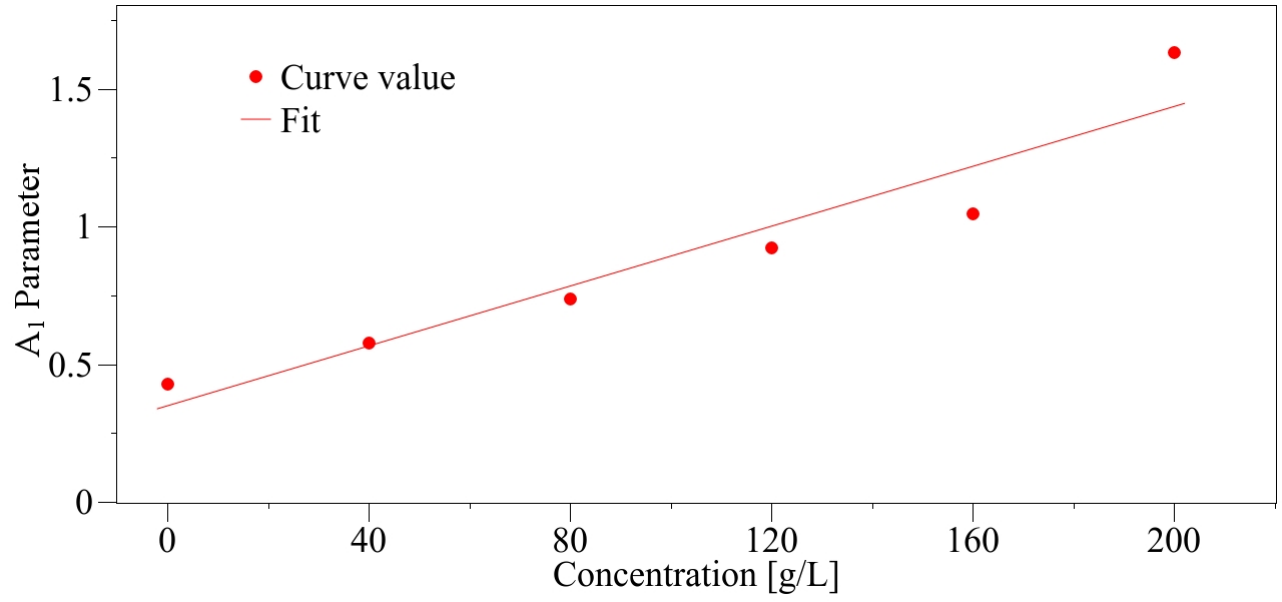
Linear relationship between the parameter *A*_1_ and the concentration.

The regression curve of the temperature *T_c_* (figure 7) is provided with *R*^2^ = 0.99 and *s* = 0.85, whereas for Δ*T*, the regression coefficient is *R*^2^ = 0.91 and the standard error is *s* = 0.028. On these calculations, the value at the concentration 200 g/L was discarded since for large salt contents the solution is inhomogeneous so that the transition is not abrupt. Additionally this shows the limitation of our method in these cases. The introduction of increasing NaCl content induces changes of the phase transition characteristics revealed by the Raman probe. First, as expected, the increase in saline water concentration diminishes the freezing point, bringing it closer to −20 °C. Simultaneously the slope Δ*T* is growing with the salt content. In the high temperature (liquid phase), a disorder increase reflected by the parameter *A*_3_ is as well observed with increasing concentration. Below the phase transition (solid phase), the plots of *S_𝒟_* are proportional to temperature (Zone II on Figure 5), so that of the parameter *A*_1_ can be easily calculated and its dependence versus the concentration gives a straight line (cf. Figure 8).

The expression of parameter *A*_1_ is thus written as:

(8) $$A_{1}=3.48\times 10^{-1}+5.43\times 10^{-4}\;\cdot \;C$$

All values and the errors associated are summarized on Table 1.

From them and from Equations 4, 7 and 8, we are able to simplify Equation 2 into Equation 9.

(9) $$S_{𝒟}=(1.16+0.0045\;\cdot \;C)+(0.00804+0.000027\;\cdot \;C)\;\cdot \;T+\frac{\left(-0.812+0.0009\;\cdot \;C)+(0.00804+0.000027\;\cdot \;C)\;\cdot \;T\right)}{1+\exp^{\frac{T-1.69+0.131\;\cdot \;C}{0.415+0.00125\;\cdot \;C}}}$$

**Figure 9.**
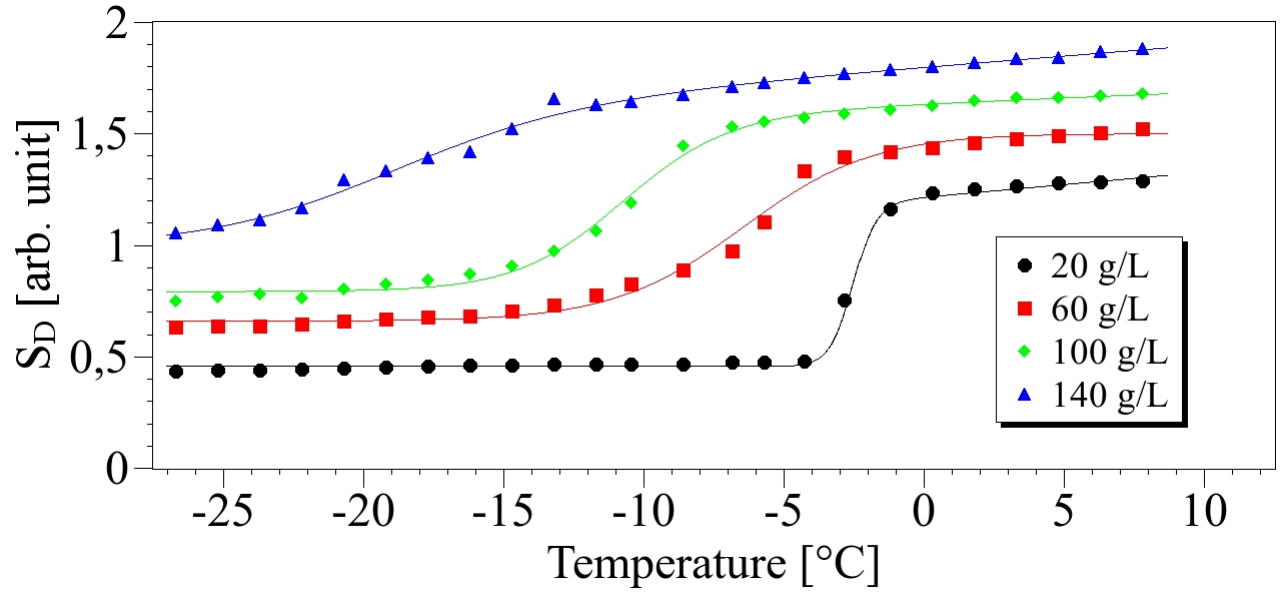
Experimental validation of the method: the curves are plots from Equation 9.

The Raman probe of the phase transition was studied for eleven saline concentrations. The resulting plots for four concentrations are shown on Figure 9.

## 4. Conclusions

We propose to use a Raman sensor to probe the liquid-solid phase transitions in salted solutions, along with its thermodynamic characteristics. This new method can be deployed on several application fields where the knowledge of the phase transition is needed. We can mention: (1) both the water and ice phases on road during winter period, (2) frozen food control, as well as its production and its storage and (3) biodiversity studies where biological cycle depends on snow/ice/water phases. Our method is based on the rapid exploitation of the *O* – *H* Raman spectrum which is found to be strongly dependent on the temperature and salt concentration. For the ratio *S_𝒟_* of two parts of the spectrum, only their integrated scattered intensities are needed without any deconvolution. Parameters describing the phase transition are derived from the quantity *S_𝒟_* and their dependences on the concentration can be easily found.

## Figures and Tables

**Table 1.** Fitted parameters of curves of figure 5.

Concentration (g/L)	Parameters				
	*A*_1_	*A*_2_	*A*_3_ (×10^−3^)	*T_c_* (°C)	Δ*T* (°C)
0	0.430 ± 0.002	1.19 ± 0.01	7.4 ± 1.5	0.01 ± 0.002	0.39 ± 0.02
40	0.580 ± 0.003	1.300 ± 0.008	11.4 ± 1.3	−5.77 ± 0.06	0.80 ± 0.05
80	0.740 ± 0.004	1.530 ± 0.007	8.8 ± 0.2	−8.23 ± 0.12	1.79 ± 0.09
120	0.920 ± 0.006	1.690 ± 0.004	10.0 ± 0.7	−9.93 ± 0.10	1.63 ± 0.09
160	1.05 ± 0.02	1.870 ± 0.002	14.6 ± 0.4	−22.26 ± 0.27	2.48 ± 0.2
200	1.64 ± 0.08	2.080 ± 0.004	12.8 ± 0.4	−25.79 ± 0.95	0.56 ± 0.05
